# DHW-221, a Dual PI3K/mTOR Inhibitor, Overcomes Multidrug Resistance by Targeting P-Glycoprotein (P-gp/ABCB1) and Akt-Mediated FOXO3a Nuclear Translocation in Non-small Cell Lung Cancer

**DOI:** 10.3389/fonc.2022.873649

**Published:** 2022-05-13

**Authors:** Mingyue Liu, Chang Xu, Xiaochun Qin, Wenwu Liu, Deping Li, Hui Jia, Xudong Gao, Yuting Wu, Qiong Wu, Xiangbo Xu, Bo Xing, Xiaowen Jiang, Hongyuan Lu, Yingshi Zhang, Huaiwei Ding, Qingchun Zhao

**Affiliations:** ^1^ Department of Life Science and Biochemistry, Shenyang Pharmaceutical University, Shenyang, China; ^2^ School of Traditional Chinese Medicine, Shenyang Pharmaceutical University, Shenyang, China; ^3^ Department of Pharmacy, General Hospital of Northern Theater Command, Shenyang, China; ^4^ School of Pharmacy, China Medical University, Shenyang, China; ^5^ Key Laboratory of Structure-Based Drug Design and Discovery of Ministry of Education, Shenyang Pharmaceutical University, Shenyang, China; ^6^ State Key Laboratory of Chemical Oncogenomics, Tsinghua Shenzhen International Graduate School, Shenzhen, China

**Keywords:** NSCLC, P-glycoprotein, FOXO3a nuclear translocation, Multidrug resistance, DHW-221, paraptosis

## Abstract

Multidrug resistance (MDR) is considered as a primary hindrance for paclitaxel failure in non-small cell lung cancer (NSCLC) patients, in which P-glycoprotein (P-gp) is overexpressed and the PI3K/Akt signaling pathway is dysregulated. Previously, we designed and synthesized DHW-221, a dual PI3K/mTOR inhibitor, which exerts a remarkable antitumor potency in NSCLC cells, but its effects and underlying mechanisms in resistant NSCLC cells remain unknown. Here, we reported for the first time that DHW-221 had favorable antiproliferative activity and suppressed cell migration and invasion in A549/Taxol cells *in vitro* and *in vivo*. Importantly, DHW-221 acted as a P-gp inhibitor *via* binding to P-gp, which resulted in decreased P-gp expression and function. A mechanistic study revealed that the DHW-221-induced FOXO3a nuclear translocation *via* Akt inhibition was involved in mitochondrial apoptosis and G0/G1 cell cycle arrest only in A549/Taxol cells and not in A549 cells. Interestingly, we observed that high-concentration DHW-221 reinforced the pro-paraptotic effect *via* stimulating endoplasmic reticulum (ER) stress and the mitogen-activated protein kinase (MAPK) pathway. Additionally, intragastrically administrated DHW-221 generated superior potency without obvious toxicity *via* FOXO3a nuclear translocation in an orthotopic A549/Taxol tumor mouse model. In conclusion, these results demonstrated that DHW-221, as a novel P-gp inhibitor, represents a prospective therapeutic candidate to overcome MDR in Taxol-resistant NSCLC treatment.

## Introduction

Non-small cell lung cancer (NSCLC) has become one of the most prevalent malignant tumors with a high incidence and fatality rate worldwide (1–3). Most NSCLC patients are diagnosed at an unresectable stage (3). Despite continuous efforts to develop efficient treatment strategies, the intrinsic and acquired resistance of chemotherapeutic agents, especially for multidrug resistance (MDR), is still a limitation for improving the efficiency of chemotherapeutics (4). Paclitaxel, a microtubule stabilizer, has become the first-line chemotherapeutic drug in the fight against advanced and metastatic NSCLC, yet acquired resistance to Taxol, including MDR, is frequently encountered, thereby leading to treatment failure and relapse (5, 6). Therefore, developing alternative therapeutic approaches is important to better understand the molecular mechanism of Taxol resistance and improve the prognosis of patients with Taxol-resistant NSCLC.

MDR in cancer is induced by various mechanisms, including the overexpression of ATP-binding cassette (ABC) transporters, enhancement of damaged DNA self-repair capacity, elevation of xenobiotics metabolisms, change of genetic factors, and abnormal activation of related signaling pathways (7, 8). P-Glycoprotein (P-gp), an ABC transporter, is encoded by ABC subfamily B member 1 (ABCB1) and is highly expressed in MDR cells, and it is responsible for decreasing drug accumulation to confer drug resistance to chemotherapeutic agents, especially to Taxol (9, 10). Therefore, it is extremely urgent to develop a novel P-gp inhibitor or antitumor drug that is not a P-gp substrate to avoid the efflux of chemotherapeutic agents and increase drug sensitivity, thereby preventing P-gp-mediated MDR.

Phosphatidylinositol-3-kinase, a lipid kinase, plays a vital role in cell proliferation, cell survival, cell cycle progression, migration, and invasion (11). More importantly, the PI3K/Akt signaling pathway is activated in cancers, including MDR tumors, and is directly involved in controlling growth regulatory transcription factors (12, 13). Forkhead box O3a (FOXO3a), a tumor suppressor in the FOXO transcription factor family (FOXO1, FOXO3, FOXO4, and FOXO6), is particularly important due to its unique role in cell apoptosis, cell cycle arrest, and longevity (14). In addition, FOXO3a, a direct downstream target of Akt, is phosphorylated by Akt at Thr32, Ser253, and Ser315, which releases it from the nucleus into the cytoplasm in an inactive form where it undergoes subsequent protease-dependent degradation (14, 15). Recently, overexpression of FOXO3a has been shown to overcome or reverse drug resistance in ovarian (16), luminal breast (17), pancreatic (18), lung (19), and colorectal (20) cancers. Consequently, blockade of the PI3K/Akt signaling pathway contributes to the activation the FOXO3a-mediated cell death progress in treating Taxol-resistant NSCLC.

Given the important role of the PI3K/Akt/mTOR signaling pathway in cancer, it has been reported that CMG002, a PI3K/mTOR dual-target inhibitor, overcomes chemoresistance in ovarian cancer (21). Additionally, studies have shown that inhibition or knockout of PI3K 110α contributes to overcome P-gp-mediated MDR in cancer (22). In a previous study, we designed and synthesized a novel PI3K/mTOR dual inhibitor, 2,4-difluoro-*N*-(5-(1-(4-(2-hydroxyethoxy)phenyl)-1*H*-benzo[*d*]imidazol-6-yl)-2-methoxypyridin-3-yl)benzenesulfonamide (DHW-221), and we demonstrated that it exerts a remarkable antitumor activity in NSCLC cells (23, 24). However, the effect of DHW-221 in Taxol-resistant NSCLC cells remains unclear. In the present study, we aimed to elucidate the molecular mechanisms of DHW-221 in overcoming MDR in A549/Taxol cells *in vitro* and *in vivo.*

## Materials and Methods

### Compounds and Reagents

DHW-221 was synthesized and purified as described previously (23). GDC-0980 and MG132 were obtained from Bide Pharmtech Ltd. (Shanghai, China). Taxol, docetaxel, vincristine, cisplatin, verapamil, and cycloheximide (CHX) were purchased from MedChem Express (NJ, USA). Rhodamine (Rho-123) and crystal violet were obtained from Shanghai Maclin Biochemical Co., Ltd. (Shanghai, China). Hoechst 33342 and 4,6-diamino-2-phenyl indole (DAPI) were obtained from Beyotime Biotechnology (Nanjing, China). The Annexin-V fluorescein isothiocyanate (FITC)/PI-double staining apoptosis detection kit and propidium iodide (PI) were obtained from Becton, Dickinson and Company (NJ, USA). P-gp antibody (CST, #13978; Rabbit, AF5185), PI3Kp110α antibody (rabbit, AF5112), p-FOXO3a (Ser253) antibody (rabbit, AF3020), and Bim antibody (Rabbit, AF0121) were purchased from Affinity Biosciences Pty Ltd. (Melbourne). Lactic dehydrogenase (LDH) detection kit (WL03271) and other primary antibodies were purchased from Wanlei Biotechnology Co., Ltd. (Shenyang, China)

### Cell Culture

Taxol-sensitive A549 cells and Taxol-resistant A549 (A549/Taxol) cells were generously gifted by Prof. Yang at Shenyang Pharmaceutical University. Cells were cultured in Roswell Park Memorial Institute (RPMI) 1640 medium supplemented with 10% fetal bovine serum (FBS) and 1% penicillin-streptomycin at 37°C and 5% CO_2_. To maintain resistant characteristics, A549/Taxol cells were cultured in medium containing 300 nM Taxol, which was replaced with drug-free culture medium for 7 days during the experiment.

### Cell Viability Assay

A 3-(4, 5-dimethylthiazol-2-yl)-2, 5-diphenyltetrazolium bromide (MTT) assay and LDH assay were selected to evaluate the effects of DHW-221 and other drugs on cell viability. First, cells were seeded into 96-well plates at 6 × 10^3^ cells/well. After cell adherence, cells were treated with various concentrations of DHW-221 and other drugs for the indicated times. For the MTT assay, cells were cultured with MTT (5 mg/ml) for 4 h. The supernatant was discarded, and the formed formazan crystals were dissolved by dimethylsulfoxide (DMSO). Absorbance was recorded at 490 nm using a microplate reader (Elx 800, Bio-Tek, Winooski, Vermont, USA ). The half-maximal inhibitory concentration (IC_50_) values were calculated by GraphPad Prism 7.0 software (San Diego, CA, USA). Importantly, the resistance index (RI) was calculated using the following formula: RI = (IC_50_ of A549/Taxol cells)/(IC_50_ of A549 cells). For the LDH assay, the cell supernatant was collected from each well and incubated with the reagent mixture according to the manufacturer’s instructions. LDH release was measured as an indicator of cytotoxicity at 450 nm by a microplate reader (Elx 800, Bio-Tek, Winooski, Vermont, USA).

### Colony Formation Assay

To evaluate the long-term antiproliferative activity of DHW-221, colony formation assay was performed as previously described (25). First, cells were seeded into six-well plates at approximately 1.5×10^3^ cells/well. After adherence, cells were treated with various concentrations of DHW-221 and GDC-0980. After 48 h, the medium was replaced with drug-free medium followed by incubation for an additional 10 days. The colonies were fixed with methanol for 10–s20 min and then stained with 0.1% (w/v) crystal violet for 30 min. Finally, 30% glacial acetic acid was used to dissolve the crystal violet, and the absorbance was measured at 570 nm by a microplate reader (Elx 800, Bio-Tek, Winooski, Vermont, USA).

### Hoechst 33342 Staining, Mitochondrial Membrane Potential (MMP) Assay, and Annexin-FITC/PI Assay

Hoechst 33342 staining, a mitochondrial membrane potential (MMP) assay, and an Annexin-FITC/PI assay were used to detect cell apoptosis. First, cells were seeded into six-well plates at 1.0 × 10^5^ cells/well and cultured overnight. Cells were then treated with various concentrations of DHW-221 and GDC-0980 for 48 h. For Hoechst 33342 staining and the MMP assay, cells were washed twice with cold phosphate-buffered saline (PBS), then stained with Hoechst 33342 or JC-1 staining solution for 15 min in the dark and then imaged using a confocal microscope (Olympus Corp., Tokyo, Japan). After Hoechst 33342 staining, normal and necrotic cells showed weak blue fluorescence, while apoptotic cells showed strong blue fluorescence. After JC-1 staining, the appearance of red and green fluorescence represented normal and decreased mitochondrial membrane potential, respectively. Of note, decreased MMP is a hallmark event in the early stages of apoptosis. For the Annexin-FITC/PI double staining assay, cells were collected, washed twice with cold PBS, and suspended in 1× binding buffer. Cells were then stained with FITC-conjugated Annexin V for 5 min, and PI was added to the cells for 15 min in the dark. Cells were then analyzed using a FACSCalibur flow cytometer (BD Biosciences, San Jose, CA, USA).

### Cell Cycle Assay

To determine the effect of DHW-221 on cell cycle distribution, PI staining was performed as described previously (25). Cells were seeded into the six-well plates at 1.0 × 10^5^ cells/well and incubated overnight. After adherence, cells were treated with various concentrations of DHW-221 and GDC-0980. After 48 h, cells were harvested and fixed in 70% cold ethanol at −20°C overnight. Next, cells were washed twice with cold PBS and stained with PI for 15 min in the dark. Finally, cells were analyzed using a FACSCalibur flow cytometer (BD Biosciences, CA, USA).

### Western Blot Analysis

Western blot analysis was used to investigate the effect of drugs on the expression levels of different proteins. First, cells were seeded into 100-mm culture dishes and incubated overnight. After treatment with various concentrations of DHW-221 and other drugs for a specified time, cells were harvested, lysed in radioimmunoprecipitation assay buffer (RIPA) buffer with 0.1 M phenylmethylsulfonyl fluoride (PMSF) and centrifuged at 13,000 rpm for 10 min at 4°C. The total protein concentration was detected using a BCA protein assay kit (Beyotime, China). The cell lysates were mixed with sample dye and boiled at 100°C for 10 min. The proteins were then resolved by sodium dodecyl sulfate–polyacrylamide gel electrophoresis (SDS-PAGE) and transferred onto polyvinylidene fluoride (PVDF) membranes. Subsequently, the PVDF membranes were blocked with 5% non-fat milk dissolved in Tris-buffered saline solution with Tween^®^ 20 (TBST) buffer for 2 h at room temperature followed by incubation with the following primary antibodies overnight at 4°C: rabbit anti-P-gp (1:1,000), anti-PI3Kp110α (1:1,000), and anti-FOXO3a (1:1,000). After incubation with peroxidase-conjugated secondary antibodies (1:12,000) for 1 h at room temperature, the protein bands were visualized with an ultrasensitive ECL chemiluminescence detection kit and analyzed by ImageJ software (National Institute of Health, MD).

### Nuclear and Cytoplasmic Fractionation

To investigate the subcellular localization of FOXO3a, the subcellular fractionation of cells was conducted with a Nuclear and Cytoplasmic Protein Extraction Kit (Wanlei, WLA020a, Shenyang, China) according to the manufacturer’s instructions. Cells were harvested, and cytoplasmic protein extraction reagent A was added followed by vortexing. Cytoplasmic protein extraction reagent B was then added. Cells were centrifuged at 12,000 rpm for 5 min at 4°C, and the resulting supernatant was the cytoplasmic protein fraction. Subsequently, the insoluble precipitates were dissolved with nuclear protein extraction reagent C and vortexed vigorously every 2 min for 30 s for eight cycles. After centrifugation at 12,000 rpm for 10 min at 4°C, the resulting supernatant was the nuclear protein fraction.

### Immunofluorescence Staining

To examine the cellular localization of certain proteins, immunofluorescence staining was performed. Cells were seeded into 24-well plates at 5 × 10^4^ cells/well and incubated overnight. After treatment with various concentrations of DHW-221 and GDC-0980, cells were fixed with 4% paraformaldehyde, permeabilized with 0.01% (v/v) Triton X-100, incubated with immune staining blocking buffer for 1 h, and then incubated at 4°C overnight with the following primary antibodies: FOXO3a, vimentin, or occludin (1:100). Cells were then incubated with FITC- or Cy3-conjugated (diluted 1:150) goat anti-rabbit IgG (H + L) for 1 h in the dark. DAPI was used to stain cell nuclei for 10 min. Finally, images were obtained with the same confocal microscope (Olympus Corp., Tokyo, Japan) and microscope settings throughout capturing of images. The fluorescence intensities of protein or fluorescent markers in the control and treatment samples were compared at the same threshold. Images were converted into grayscale images with ImageJ software, and positive pixel area (PPA) was analyzed by applying the same threshold to measure the mean fluorescence intensity.

### Wound-Healing Assay and Transwell Assay

A wound-healing assay and a Transwell assay were performed to assess the effect of DHW-221 treatment on the migration and invasion capability of A549/Taxol cells. For the wound-healing assay, cells were seeded into six-well plates at 2.0 × 10^5^ cells/well and incubated overnight. A 200-μl pipette tip was used to scratch a straight line through the monolayer on the bottom of the six-well plate. After treatment with various concentrations of DHW-221 and GDC-0980 for 24 h, images were acquired using a microscope and subsequently imported into ImageJ software to evaluate the wound area. For the Transwell assay, serum-free medium containing 1.0 × 10^5^ A549/Taxol cells (200 μl) was placed into the upper chamber of a 24-well Transwell plate (Corning Life Sciences, Bedford, MA, USA) with or without Matrigel, and cells were treated with DHW-221 (0.05, 0.10, and 0.15 µM) and GDC-0980 (0.10 µM). Medium containing 10% FBS (600 μl) was used as a chemoattractant in the lower chamber. After 24 h, the invaded cells in the lower chamber were fixed with methanol, stained with 0.1% crystal violet solution, and imaged using a microscope. Finally, 33% glacial acetic acid solution was added to the lower chamber, which was shaken for 10 min. Absorbance was then measured at 570 nm using a microplate reader (Elx 800, Bio-Tek, Winooski, Vermont, USA).

### Molecular Docking

To predict the potential binding mode of DHW-221 and ABCB1, molecular docking was performed. The crystal structures of the human ABCB1-tariquidar complex (PDB code: 7A6E) and ABCB1-ATP complex (PDB code: 6C0V) were downloaded from Protein Data Bank (http://www.rcsb.org). The Protein Preparation Wizard in Maestro (version 11.5) was utilized to remove water, add hydrogenation, and prime. The DHW-221 was drawn, saved as a “mol.” format by ChemDraw14.0 Ultra software, and optimized with the LigPrep module. The Grid Generation Tool was then used to define the binding pocket (20 Å radius) of ABCB1. Finally, the predicted binding conformations of ABCB1-DHW-221 were generated to select the optimal binding mode using the Ligand Docking module and PYMOL 2.5 software. The co-crystallized tariquidar ligand and ATP were selected as templates for molecular docking.

### Cellular Thermal Shift Assay

Cellular Thermal Shift Assay (CETSA) was conducted to explore target engagement of DHW-221 to ABCB1 in A549/Taxol cells. Cells were seeded into 100-mm cultural dishes. After reaching 80%–90% confluency, cells were treated with DMSO, DHW-221 (0.60 µM), or verapamil (0.60 µM) for 3 h. Cells were harvested with a cell scraper, resuspended in cold PBS, and equally divided into six parts into 0.2-ml PCR tubes (80 µl/tube). To lyse cells, six repeated cycles of freeze–thawing (37°C and 57°C) were performed using liquid nitrogen. After centrifugation at 14,000*g* for 30 min at 4°C, 50 µl of the soluble fraction was removed, mixed with SDS-sample buffer (5´), and heated in boiling water for 10 min. The samples were then evaluated by Western blot analysis.

### Intracellular Rho-123 Uptake

Intracellular Rho-123 uptake was performed to evaluate the effect of DHW-221 on P-gp function. Cells were seeded into six-well plates at 1.0 × 10^5^ cells/well and incubated overnight. After treatment with DHW-221 or verapamil for 48 h, Rho-123 (15 µM) was added into the medium for co-treatment with DHW-221 or verapamil for 3 h in the dark. Cells were then washed twice with cold PBS, fixed with 4% paraformaldehyde for 30 min, permeabilized with 0.1% Triton X-100, and incubated with DAPI for 15 min. Images were acquired using a confocal microscope (Olympus Corp., Tokyo, Japan). Cells were then harvested, washed twice with cold PBS, and resuspended to detect Rho-123 accumulation by FACSCalibur flow cytometer (BD Biosciences, San Jose, CA, USA).

### Human Orthotopic A549/Taxol Mouse Tumor Model

**Figure 1:**
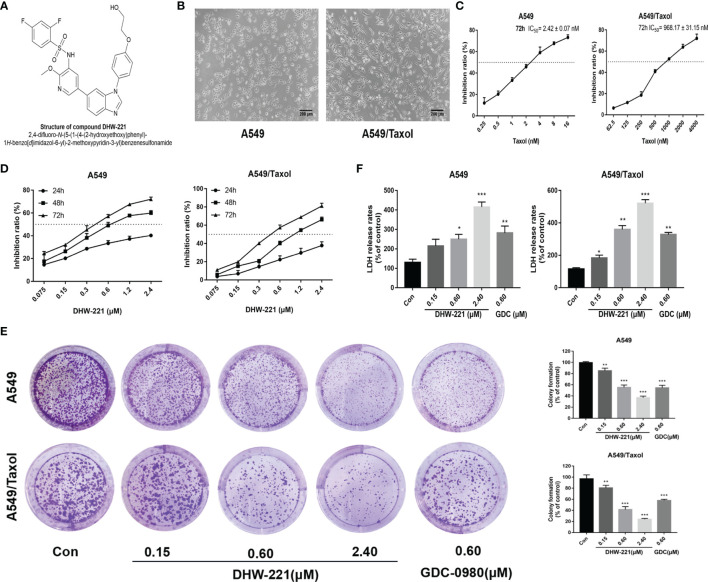
DHW-221 inhibits cell viability in A549 and A549/Taxol cells. **(A)** The chemical structure of DHW-221h. **(B)** Representative morphology of parental and Taxol-resistant NSCLC cells (A549 and A549/Taxol cells). Scale bar = 200 μm. **(C)** Inhibition effect of A549 and A549/Taxol cells after exposure to Taxol for 72 h. **(D)** Cell viability of A549 and A549/Taxol cells treated with various concentrations of DHW-221 for 24,48, and 72 h. **(E)** The growth-inhibitory effect of DHW-221 on A549 and A549/Taxol cells was measured by colony formation assay; the quantitative results were illustrated as the right panel. **(F)** Cytotoxicity of DHW-221 was examined by LDH release assay for 48 h. Statistical comparisons were performed with one-way ANOVA followed by Dunnett’s *post-hoc* test for multiple comparisons (n = 3). Data were expressed as mean ± SD. *p < 0.05, **p < 0.01, ***p < 0.001 versus control.

To evaluate the antitumor activity of DHW-221 in Taxol-resistant lung neoplasm, we established a human orthotopic nude mouse model for Taxol-resistant lung cancer *via* tail vein injection. Male nude mice (4–6 weeks old and 18–20 g) were obtained from Beijing Weitonglihua Laboratory Animal Technology Co., Ltd. (Beijing, China, and USA) and fed in specific pathogen-free (SPF) conditions. Specifically, A549/Taxol cells (2.0 × 10^6^/200 µl, per mice) were resuspended in cold PBS and inoculated into nude mice *via* tail vein injection. After 7 days, the tumor-bearing nude mice were randomly divided into six groups as follows: model (vehicle, CMC-Na + 1% Tween-80 + 0.3% DMSO), Taxol (10 mg/kg, Taxol injection diluted in saline), DHW-221 (10, 20, and 40 mg/kg, dissolved in the same vehicle as the model group), and GDC-0980 (10 mg/kg, dissolved in the same vehicle as the model group). The nude mice in the Taxol group were intraperitoneally administered with Taxol every 2 days, while the nude mice in the model, DHW-221, and GDC-0980 groups were intragastrically administered daily for 2 weeks. The body weights of the mice in each group were monitored every day. Finally, the mice were sacrificed, and the visceral organs (heart, liver, spleen, lung, and kidney) were removed, weighed, and used for further experiments. Bouin’s solution was used to stain the lung tissues. The other visceral organs were fixed with 4% paraformaldehyde and stored at room temperature for subsequent experiments. The structure of compound DHW-221 was shown in **Figures 1A**.

### Hematoxylin and Eosin Staining and Immunohistochemistry

To examine the histological and pathological changes of DHW-221 in Taxol-resistant lung neoplasm, HE staining and immunohistochemistry (IHC) were performed. All tissues were fixed with 4% paraformaldehyde, dehydrated in a graded ethanol solution (70%, 80%, 90%, 95%, and 100%, v/v), incubated in xylene until transparent, and embedded in paraffin. Subsequently, the tissues were cut into approximately 5-µm-thick sections for further study. For pathological examination, paraffin sections were sequentially deparaffinized in a xylene (xylene I, xylene II, and xylene III) and graded alcohol solution (90%, 80%, and 70%, v/v). Paraffin sections were stained with hematoxylin and eosin (HE) to distinguish tumorous metastatic foci and normal tissues. For IHC, the paraffin sections were incubated with the following primary antibodies: anti-Ki67, anti-FOXO3a, and anti-p-FOXO3a. The sections were then incubated with the appropriate secondary antibody according to the DAB kit. Finally, images were acquired using a Keyence BZ-X700 microscope and BZ-X analyzer software (Osaka, Japan). Brown granules were considered as positive staining in the cytoplasm or nucleus.

### Evaluation of Serum Biochemical Parameters

Serum biochemical parameters were used to evaluate drug toxicity according to the manufacturer’s protocols using the corresponding kits obtained from Nanjing Jiancheng Biochemical Co., Ltd. (Nanjing, China). Creatine kinase (CK), creatinine (CRE), alanine aminotransferase (ALT), and aspartate aminotransferase (AST) were evaluated to analyze heart, kidney, and liver functions, respectively. Blood was collected from the mouse eye socket and incubated for 1 h at 37°C. After centrifugation twice at 3,500 rpm for 15 min at 4°C, the supernatant (serum) was collected and stored at −80°C. Next, the serum (5 μl) was added to 96-well plates, mixed with different reagents, and incubated for the indicated time according to the manufacturer’s instructions. The absorbance was measured at 510 nm using a microplate reader. The ALT, AST, CRE, and CK values in serum were calculated using a standard curve in the manufacturer’s instructions.

### Statistical Analysis

All experiments were repeated in triplicate. Data are presented as mean ± SD. Statistical analysis was performed using GraphPad Prism 7.0 software (San Diego, CA, USA). Data were analyzed with one-way ANOVA followed by Dunnett’s *post-hoc* test for multiple comparisons. Unpaired Student’s t-test was used to compare the difference between two experimental groups. p < 0.05 was considered as statistically significant.

## Results

### DHW-221 Exerts Significant Inhibitory Activity in MDR Cancer Cells

Previously, we reported that DHW-221 exerts a remarkable antitumor activity in NSCLC cells (24), but the effect of DHW-221 in MDR NSCLC cells remains unclear. To determine whether A549/Taxol cells are MDR, we compared the cellular morphological changes in A549/Taxol and A549 cells. As depicted in **Figures 1B, C**, A549/Taxol cells were fibrous and elongated with a clear boundary, while A549 cells were cobblestone-like and clustered. In addition, the MTT assay indicated that A549/Taxol cells were resistant to Taxol, docetaxel, vincristine, and cisplatin with the resistance index (RI) values of 403.4, 392.2, 20.5, and 14.2, respectively (**Table 1**). These findings indicated that the A549/Taxol cells were MDR. Next, to evaluate the effect of DHW-221 on the cell viability of A549/Taxol and A549 cells, an MTT assay was performed. Our results showed that DHW-221 exhibited significant cytotoxicity in a concentration- and time-dependent manner in A549/Taxol and A549 cells with an RI of 1.2 (**Figure 1D** and **Table 1**). Of note, the IC_50_ values are shown in **Supplementary Table S1**. Although there was significant inhibitory activity of DHW-221 between 48 and 72 h in both cells (**Supplementary Figure S1**), the tumor cells within 48 h were in the optimal stage of logarithmic growth phase. Hence, we selected the 0.15, 0.60, and 2.40 μM concentrations and 48 h as the optimized conditions for subsequent experiments in A549/Taxol and A549 cells. In addition, colony formation assay was performed to assess the long-term antiproliferative activity of DHW-221. The results demonstrated that DHW-221 decreased the colony formation ability of A549/Taxol and A549 cells in a concentration-dependent manner (**Figure 1E**). Furthermore, an LDH release assay was used to evaluate the cell toxicity of DHW-221, and the results showed that DHW-221 treatment resulted in severe cellular damage as compared to control group in both cells (**Figure 1F**). The above results demonstrated a better antiproliferative activity of DHW-221 (48 h—A549: IC_50_ = 0.631 ± 0.072 μM; A549/Taxol: IC_50_ = 0.874 ± 0.056 μM) than that of GDC-0980 (48 h—A549: IC_50_ = 0.864 ± 0.033 μM; A549/Taxol: IC_50_ = 0.975 ± 0.026 μM), which is a dual PI3K/mTOR inhibitor and was used as a positive control (**Figure 1F** and **Supplementary Figure S1**). These results revealed that DHW-221 exerts a significant inhibitory activity in MDR cancer cells.

**Table 1:** The resistance index (RI) for various compounds at 72 h in A549/Taxol cells (mean ± SD, n = 3).

Comp.	IC_50_ (μM)		RI
	A549	A549/Taxol	
Docetaxel	0.0012 ± 0.0002	0.4706 ± 0.0891	392.2
Vincristine	0.0021 ± 0.0008	0.0430 ± 0.0011	20.5
Cisplatin	5.6645 ± 1.1759	80.2240 ± 1.0672	14.2
DHW-221	0.4242 ± 0.0238	0.5274 ± 0.0339	1.2

### DHW-221 Acts as a P-gp Inhibitor in A549/Taxol Cells Through Inhibiting P-gp Function and Expression

**Figure 2:**
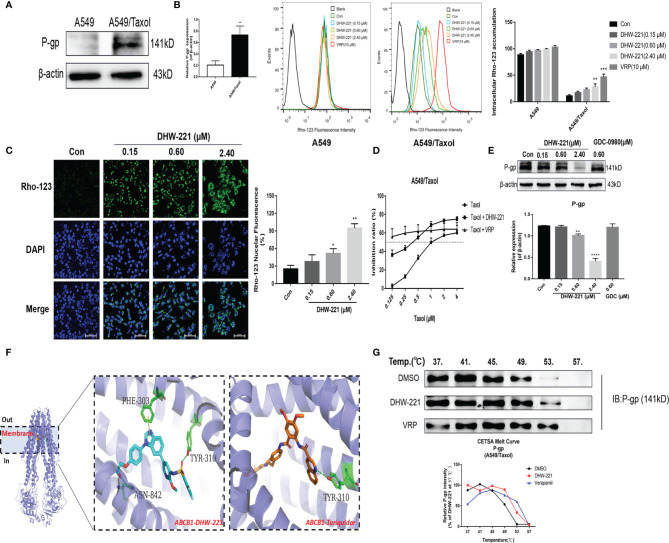
DHW-221 accelerates intracellular Rho-123 accumulations and inhibits P-gp expression by binding to P-gp. **(A)** Comparison of P-gp in A549 and A549/Taxol cells. Statistical comparisons were performed with unpaired Student’s t-test (n = 3). **p < 0.01 versus A549. **(B, C)** After treatment of DHW-221 or verapamil for 48 h, Rho-123 (15 µM) was added into the mediums to co-incubate with DHW-221 or verapamil for 3 h in dark. The intracellular accumulation of Rho-123 was evaluated by flow cytometry and confocal microscope, respectively. Verapamil (VRP, 10 μM) was used as a positive control. Green fluorescence indicated Rho-123, and blue fluorescence indicated DAPI. Scale bar = 100 μm. Nuclear mean fluorescence intensity of Rho 123 was measured by ImageJ and analyzed using FlowJo 10 software. Statistical comparisons were performed with one-way ANOVA followed by Dunnett’s *post-hoc* test for multiple comparisons (n = 3). *p < 0.05, **p < 0.01, ***p < 0.001 versus control. **(D)** Effect of DHW-221 or verapamil to reverse Taxol resistance for 48 h in A549/Taxol cells. **(E)** Western blot analysis of DHW-221 inhibited P-gp expression in A549/Taxol cells. **(F)** Ribbon diagram of human ABCB1 (PDB code: 7A6E, purple spiral) bound to drug (membrane region) and predicted binding modes for DHW-221 (blue stick) and tariquidar (orange stick, a known third-generation P-gp inhibitor) with ABCB1. Hydrogen bonds were shown as green dashed lines. Key residues for DHW-221 and tariquidar interaction were highlighted. **(G)** Effect of DHW-221 on the thermal stability of ABCB1 was quantitatively detected by a cellular thermal shift assay (CETSA). Statistical comparisons were performed with one-way ANOVA followed by Dunnett’s *post-hoc* test for multiple comparisons (n = 3). Data are presented as mean ± SD. **p < 0.01, ****p < 0.0001versus control.

Overexpression of P-gp is the most common cause of MDR in cells, and it causes a decrease in the intracellular concentration of chemotherapeutic drugs (9). To confirm whether A549/Taxol cells exhibit clinically relevant drug resistance, Western blot analysis was performed to detect P-gp expression in A549/Taxol cells. Western blot analysis showed that A549/Taxol cells exhibited a higher expression of P-gp than A549 cells (**Figure 2A**). As a yellow-green fluorescence substrate of P-gp, Rhodamine 123 (Rho-123) directly reflects P-gp function according to cellular accumulation (26). To determine whether DHW-221 affects P-gp function, FACS analysis was performed to detect the intracellular accumulation of Rho-123. As shown in **Figure 2B**, the intracellular accumulation of Rho-123 in A549/Taxol cells was significantly lower than that in A549 cells. Verapamil (VRP), a known first-generation P-gp inhibitor, was used as a positive control. Cotreatment with DHW-221 and verapamil significantly enhanced the intracellular Rho-123 accumulation in A549/Taxol cells without affecting A549 cells. Furthermore, fluorescence microscopy analysis demonstrated that the green fluorescence intensity was significantly increased in A549/Taxol cells, further confirming that DHW-221 increases intracellular Rho-123 accumulation in a concentration-dependent manner in A549/Taxol cells (**Figure 2C**). These results suggested that DHW-221 inhibits P-gp function through increasing the intracellular accumulation of Rho-123 in A549/Taxol cells.

In recent years, the development of novel P-gp inhibitors has been regarded as a promising strategy to overcome MDR (27, 28). To explore whether DHW-221 acts as a P-gp inhibitor, we examined the cell growth inhibitory effect of Taxol alone and in combination with DHW-221 or verapamil in A549/Taxol cells. Here, we selected 50 nM DHW-221 and 10 μM verapamil, which had no significant cytotoxicity in A549/Taxol cells (**Figure 1D**). The cell viability assay showed that cotreatment with DHW-221 and Taxol enhanced the cytotoxic effect of A549/Taxol cells, which was similar to the effects of verapamil, suggesting that DHW-221 functions as a P-gp inhibitor and may be a MDR reversal agent (**Figure 2D**). Moreover, the expression of P-gp was significantly downregulated in DHW-221-treated A549/Taxol cells (**Figure 2E**). These results demonstrated that DHW-221 acts as a P-gp inhibitor through inhibiting P-gp function and protein expression.

Based on the inhibitory activity of DHW-221 on P-gp, we performed molecular docking and CETSA to further elucidate whether DHW-221 binds to P-gp. The active pocket of P-gp mainly contains the transmembrane (TM, including the drug-binding site) and ATP-binding domains (29). In order to determine whether DHW-221 bind to the drug binding site of P-gp, molecular docking was performed. As shown in **Figure 2F**, DHW-221 fitted well into the drug-binding site of P-gp with a relatively higher binding affinity than tarquidar (a third-generation P-gp inhibitor), and it formed H-bonds with Phe303, Asn842, and Tyr310 residues. The glide scores of DHW-221 and tarquidar were −12.246 and −11.482 kcal/mol, respectively. These results suggested that DHW-221 binds to P-gp in the TM region, thereby reducing drug efflux. Meanwhile, 6C0V protein model was selected to evaluate whether DHW-221 bind to the ATP binding pocket of P-gp. We found that DHW-221 possessed a weaker affinity (−5.646 kcal/mol) compared to the drug-binding site (data not shown). Additionally, CETSA demonstrated that DHW-221 treatment enhanced the thermal stability of ABCB1 protein, similar to the effect of verapamil (**Figure 2G**). Taken together, these results suggested that DHW-221 inhibits P-gp function and expression through binding to the TM region of P-gp.

### DHW-221 Triggers Apoptosis and Paraptosis Through the Mitochondrial Pathway, ER Stress, and MAPK Signaling Pathway in A549/Taxol Cells

**Figure 3:**
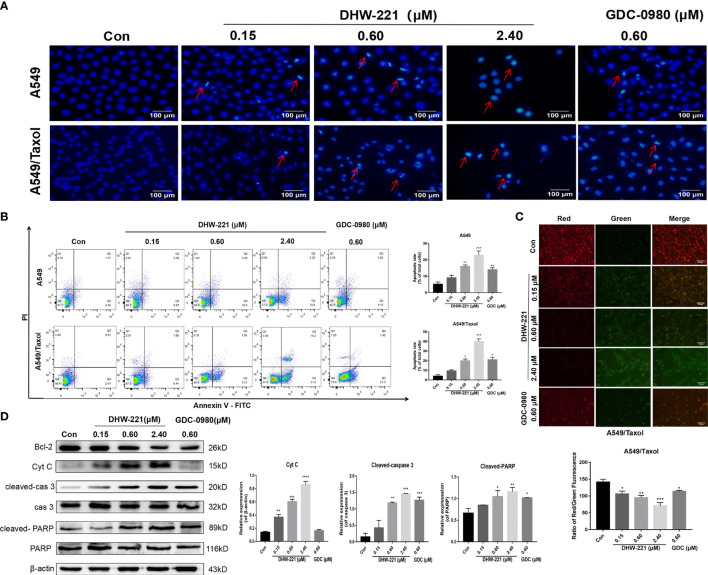
DHW-221 exerts an evident pro-apoptotic effect through the mitochondrial pathway in A549/Taxol cells. **(A)** Hoechst 33342 staining results for 48 h in A549 and A549/Taxol cells. Strong blue fluorescence indicated apoptotic cells, scale bar = 100 µm. **(B)** Induction of apoptosis by various concentrations of DHW-221 in A549 and A549/Taxol cells were evaluated by Annexin-V-FITC/PI staining for 48 h. **(C)** The changes of mitochondrial membrane potential in A549/Taxol cells were detected by JC-1 staining after exposure to DHW-221 and GDC-0980. Green and red fluorescence represented decreased and normal mitochondrial membrane potential, respectively. Scale bar = 100 µm. The red and green mean fluorescence intensity were measured by ImageJ software, and the relative radio was analyzed using GraphPad Prism 7.0 software. Statistical comparisons were performed with one-way ANOVA followed by Dunnett’s *post-hoc* test for multiple comparisons (n = 3). *p < 0.05, **p < 0.01, ***p < 0.001 versus control. **(D)** Western blot analysis of apoptosis-related proteins in A549/Taxol cells. Cells were treated with various concentrations of DHW-221 (0.15, 0.60, and 2.40 µM) and GDC-0980 (0.60 µM) for 48 h. Statistical comparisons were performed with one-way ANOVA followed by Dunnett’s *post-hoc* test for multiple comparisons (n = 3). Data were expressed as mean ± SD. *p < 0.05, **p < 0.01, ***p < 0.001, ****p < 0.0001 versus control.

Previous studies have shown that DHW-221 has a pro-apoptotic effect in NSCLC cells (24). Therefore, we investigated whether DHW-221 triggers apoptosis in A549/Taxol cells. Hoechst 33342 staining was performed to detect the cellular morphological changes in each group. We observed condensed chromatin and smaller nuclei accompanied with strong blue fluorescence in both A549/Taxol and A549 cells (**Figure 3A**). Next, A549/Taxol and A549 cells were treated with various concentrations of DHW-221 and GDC-0980, and cell apoptosis was assessed by FACS analysis. Compared to the control group, the rates of early and late apoptosis were significantly increased in the DHW-221-treated group in both A549/Taxol and A549 cells (**Figure 3B**). At a concentration of 2.4 μM, the pro-apoptotic ability of DHW-221 in A549/Taxol cell was stronger than that of A549 cells (**Figure 3B**), which agreed with previous results (**Figure 1D**). These results demonstrated that DHW-221 triggers apoptosis in A549/Taxol and A549 cells in a concentration-dependent manner. To elucidate the underlying mechanism of DHW-221-triggered apoptosis in A549/Taxol cells, we examined the MMP by JC-1 staining. A decrease in the MMP is a landmark event in the early stage of apoptosis. After treatment with DHW-221 and GDC-0980, JC-1 did not exist in the mitochondrial matrix in the form of polymers, resulting in significantly reduced red fluorescence intensity in the mitochondria and significantly enhanced green fluorescence in the cytoplasm (**Figure 3C**), which indicated that the intracellular MMP was decreased by DHW-221 in A549/Taxol cells. These results implied that cell apoptosis induced by DHW-221 may occur *via* the mitochondrial pathway. We next investigated the expression of mitochondrial apoptosis pathway-related proteins by Western blot analysis. In a concentration-dependent manner, DHW-221 upregulated the expression levels of apoptosis-related proteins [cytochrome C, cleaved caspase 3, and cleaved poly(ADP-ribose) polymerase (PARP)] and downregulated the expression levels of the anti-apoptotic protein, Bcl-2, in A549/Taxol cells (**Figure 3D**). These results demonstrated that apoptosis induced by the mitochondrial pathway participates in DHW-221-induced cell death in A549/Taxol cells.

**Figure 4:**
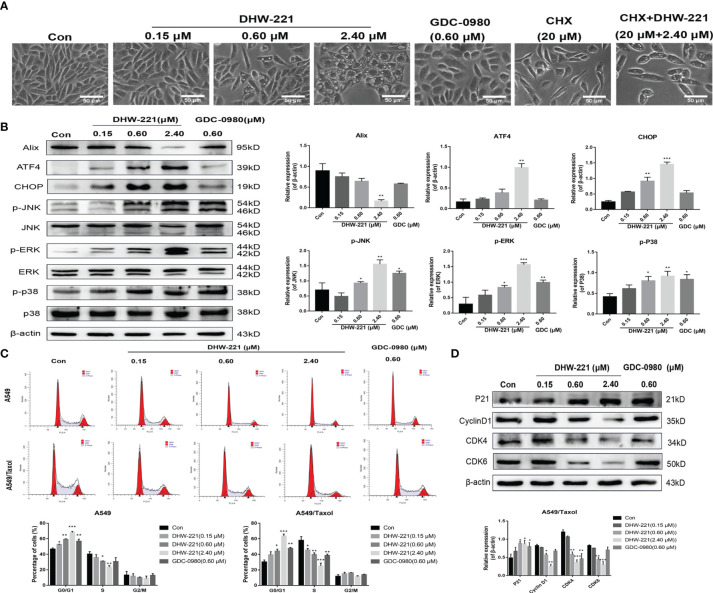
DHW-221 induces paraptosis and cell cycle arrest in A549/Taxol cells. **(A)** Representative microscope images of DHW-221-incubated A549/Taxol cells. Cells were pretreated with cycloheximide (CHX, 20 μM) followed by DHW-221 (2.40 μM) treatment for 12 h. Scale bar = 50 µm. **(B)** The expression levels of Alix, ER stress (ATF4 and CHOP) and MAPK signaling pathway related proteins (p-ERK, p-JNK, and p-P38) in A549/Taxol cells were measured by Western blot. The histogram (below) indicated the relative band intensity ratio of each protein for three independent experiments. (h) **(C)** The cell cycle distribution followed by DHW-221 and GDC-0980 treatment for 48 h in A549/Taxol cells was illustrated by FACS analysis. **(D)** The expression levels of cell cycle-associated proteins were measured by Western blot. The relevant quantitative results were shown in the figure below. Statistical comparisons were performed with one-way ANOVA followed by Dunnett’s *post-hoc* test for multiple comparisons (n = 3). Data are presented as mean ± SD. *p < 0.05, **p < 0.01, ***p < 0.001 versus control.

Interestingly, we observed that high-concentration DHW-221 exacerbated the formation of massive vacuoles in A549/Taxol cells (**Figure 4A**). As cytoplasmic vacuolization is strongly linked to paraptosis-like cell death (30), we hypothesized that DHW-221 may induce paraptosis in A549/Taxol cells. Paraptosis is a non-apoptotic and caspase-independent form of programmed cell death that is completely different from apoptosis and requires protein synthesis (31). To test our hypothesis, the protein synthesis inhibitor, cycloheximide (CHX), was added to DHW-221-treated A549/Taxol cells. Cotreatment with CHX and DHW-221 attenuated cytoplasmic vacuolation compared to treatment with 2.4 μM DHW-221 alone (**Figure 4A**). Furthermore, Western blot analysis showed that DHW-221 significantly inhibited the expression of Alix, a well-known paraptosis regulator that inhibits the onset of paraptosis (**Figure 4B**). Some studies have shown that paraptosis-like cell death induced by Zika virus (ZIKV) or ^125^I radioactive seeds is regulated by the PI3K/Akt signaling pathway (32, 33). In addition, the mTOR inhibitor, everolimus, triggers cell paraptosis in childhood acute lymphoblastic leukemia (34). Based on these studies, our findings suggested that DHW-221 increases the pro-paraptotic ability of A549/Taxol cells. Because the process of paraptosis is inseparable from the involvement of endoplasmic reticulum (ER) stress and mitogen-activated protein kinase (MAPK) signaling pathway (35), we evaluated the protein expression of key regulatory proteins in ER stress and the MAPK signaling pathway by Western blot analysis. As shown in **Figure 4B**, the expression levels of ATF4, CHOP (key regulator in ER stress), p-JNK, p-ERK, and p-p38 were significantly upregulated after treatment with DHW-221. These findings indicated that activated ER stress and the MAPK signaling pathway lead to DHW-221-triggered paraptosis in A549/Taxol cells.

### DHW-221 Induces Cell Cycle Arrest at the G0/G1 Phase in A549/Taxol Cells

Considering that PI3K inhibitors eliminate tumor cells by inducing G0/G1 phase arrest in various solid tumors (36, 37), we investigated whether DHW-221 exerts a similar effect in A549/Taxol cells by evaluating the effect of DHW-221 on cell cycle distribution by FACS analysis. Our results showed that DHW-221 treatment for 48 h significantly reduced the proportion of cells in S phase and increased the proportion of cells in G0/G1 phase, indicating that DHW-221 arrests the cell cycle at the G0/G1 phase in A549/Taxol and A549 cells (**Figure 4C**). To further elucidate the potential mechanism of DHW-221-induced cell cycle arrest, we examined the expression levels of key G0/G1 phase-related proteins by Western blot analysis. The cyclin D1-CDK4 complex activity is regulated by p21 and is the key to block cancer cells entering into S phase (38). **Figure 4D** shows that DHW-221 treatment decreased the expression levels of cyclin D1, CDK4, and CDK6 and increased the expression levels of p21 in a concentration-dependent manner. These data suggested that G0/G1 cell cycle arrest is involved in DHW-221-induced cell death in A549/Taxol cells.

### Akt-Mediated FOXO3a Nuclear Translocation Is Involved in Mitochondrial Apoptosis and G0/G1 Cell Cycle Arrest Induced by DHW-221 in A549/Taxol Cells

**Figure 5:**
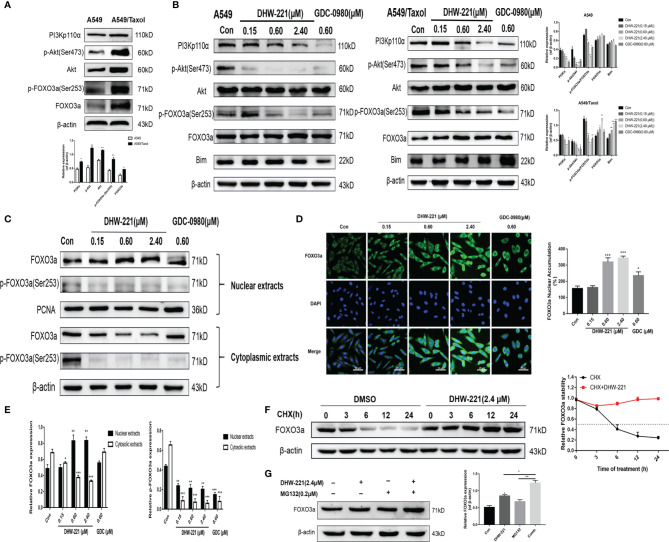
DHW-221-triggered mitochondrial apoptosis is linked to Akt-mediated FOXO3a nuclear translocation in A549/Taxol cells. **(A, B)** Comparison and the expression levels of PI3K/Akt/FOXO3a signaling pathway related proteins in A549 and A549/Taxol cells were determined by Western blot, in the presence and absence of DHW-221 and GDC-0980. Statistical comparisons were performed with unpaired Student’s t-test (n = 3) in Panel **(A)**. *p < 0.05, **p < 0.01 versus A549. Statistical comparisons were performed with one-way ANOVA followed by Dunnett’s *post-hoc* test for multiple comparisons in Panel **(B)** (n = 3). *p < 0.05, **p < 0.01, ***p < 0.001 versus control. **(C, E)** The FOXO3a and p-FOXO3a expressions in the nuclear and cytoplasmic fractions of A549/Taxol cells were detected by Western blot. Proliferating cell nuclear antigen (PCNA) was selected as nucleoprotein internal control. The quantitative results were shown in Panel **(E)**. **(D)** Immunofluorescence staining of FOXO3a in A549/Taxol cells was carried out to evaluate the effect of DHW-221 on FOXO3a nuclear translocation. Scale bar = 20 µm. The histograms indicated the percentage of the cells in each condition exhibiting FOXO3a nuclear mean fluorescence intensity (positive cells, green fluorescence) by ImageJ. **(F)** FOXO3a degradation in A549/Taxol cells with or without DHW-221 co-treatment in different time spot when protein biosynthesis was blocked with 20 µM cycloheximide (CHX). FOXO3a stability was analyzed relative to control by ImageJ software. **(G)** FOXO3a proteins levels in the presence of MG132 (0.20 μM) or 2.40 μM DHW-221-treated A549/Taxol cells at 24 h. Statistical comparisons were performed with one-way ANOVA followed by Dunnett’s *post-hoc* test for multiple comparisons (n = 3). Data were presented as mean ± SD. *p < 0.05, **p < 0.01, ***p < 0.001 versus control.

Aberrant activation of some signaling pathways, including the PI3K/Akt signaling pathway, has been recognized as a common cause of MDR (39). Western blot analysis demonstrated that the PI3K/Akt/FOXO3a signaling pathway was hyperactivated in A549/Taxol cells compared to A549 cells (**Figure 5A**). Extensive studies have shown that blocking the PI3K/Akt signaling pathway enhances the sensitivity of various drug-resistant human cancer cells to several drugs, including cisplatin, oxaliplatin, and gemcitabine (39–41). Thus, we assessed the effect of DHW-221 on the PI3K/Akt signaling pathway in A549/Taxol and A549 cells by Western blot analysis. Treatment with various concentrations of DHW-221 and GDC-0980 significantly decreased the expression levels of PI3Kp110α and p-Akt in a concentration-dependent manner, while no obvious change was observed in the total Akt level, which suggested that DHW-221 blocked the PI3K/Akt signaling pathway in both A549/Taxol and A549 cells (**Figure 5B**), agreeing with a previous study (24). FOXO3a, a direct downstream target of Akt, is directly phosphorylated by Akt at the Ser253 site, which allows it to bind to its chaperone protein, 14-3-3, resulting in transfer from the nucleus to the cytoplasm; this process is an important tumorigenic mechanism for evading cancer cell apoptosis (14). In addition, the pro-apoptotic protein, Bim, is regulated by FOXO3a at the transcriptional level (42). To investigate whether DHW-221-induced cell apoptosis is mediated *via* the Akt/FOXO3a/Bim pathway, we utilized Western blot analysis. In A549/Taxol cells, DHW-221 treatment significantly downregulated p-FOXO3a (Ser253) expression and upregulated FOXO3a expression, and these changes were accompanied by an increase in Bim expression. In contrast, no significant changes in FOXO3a and Bim expression levels were observed in DHW-221-treated A549 cells, indicating that DHW-221-induced cell apoptosis is unrelated to FOXO3a expression in A549 cells. However, these findings need to be further confirmed by substantial experiments in future work.

To further investigate the relocalization of FOXO3a in DHW-221-treated A549/Taxol cells, Western blot analysis and immunofluorescence staining were performed to examine the subcellular localization of FOXO3a. As shown in **Figures 5C, E**, DHW-221 increased nuclear FOXO3a protein and decreased cytoplasmic phosphorylated FOXO3a at the Ser253 site, which indicated that FOXO3a was predominantly transferred from the cytoplasm to the nucleus by DHW-221 in A549/Taxol cells. Furthermore, DHW-221 caused nuclear translocation of FOXO3a, which was manifested by a gradual increase in green fluorescence intensity in the nucleus (**Figure 5D**). These results suggested that DHW-221 induces Akt-mediated FOXO3a nuclear translocation. Studies have shown that FOXO3a directly affects the expression of the downstream target genes, p21 and cyclin D1 (42), which was in agreement with the results of the present study (**Figure 4D**). These results suggested that DHW-221-induced mitochondrial apoptosis and cell cycle arrest are closely related to Akt-mediated FOXO3a nuclear translocation in A549/Taxol cells.

To elucidate the mechanism of the DHW-221-mediated increase in FOXO3a expression, CHX and the proteasome inhibitor, MG132, were added into culture media to inhibit protein synthesis. We first measured the half-life of FOXO3a after exposure to CHX alone and in combination with DHW-221 at various time points in A549/Taxol cells by Western blot analysis. As shown in **Figure 5F**, FOXO3a expression was attenuated by CHX in the DMSO group, whereas cotreatment of DHW-221 and CHX restored FOXO3a expression, which indicated that DHW-221 prevents FOXO3a degradation, thus increasing FOXO3a stability. **Figure 5G** shows that FOXO3a expression was slightly enhanced upon MG132 treatment, which suggested that FOXO3a was instable, indicating that the combination of DHW-221 and MG132 unexpectedly promoted FOXO3a accumulation in A549/Taxol cells. These results demonstrated that DHW-221 interferes with FOXO3a degradation in a proteasome-independent manner.

### DHW-221 Suppresses Migration and Invasion Through Inhibiting Epithelial-Mesenchymal Transition Phenotypic Changes

**Figure 6:**
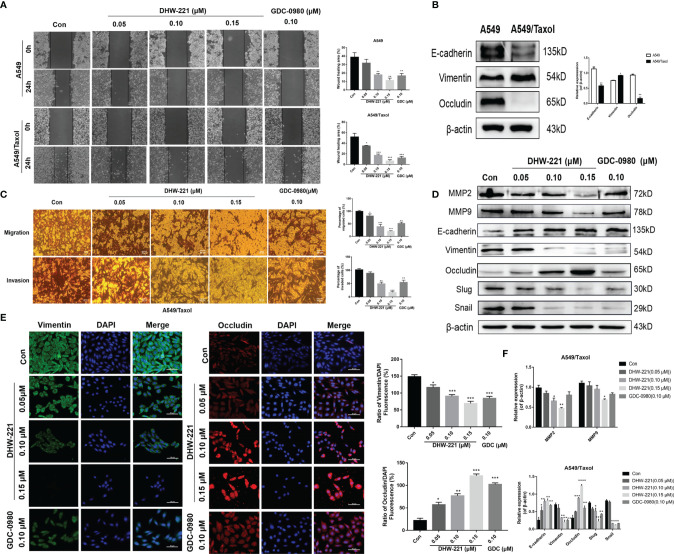
Cell migration and invasion caused by EMT phenotype are inhibited by DHW-221 in A549/Taxol cells. **(A, C)** The effects of various concentrations of DHW-221 on the migration and invasion of A549/Taxol cells were studied by the wound healing assay and Transwell assays, respectively. The corresponding quantitative statistical graphs were attached to the side. **(B)** The changes of EMT markers (E-cadherin, vimentin, and occludin) in A549 and A549/Taxol cells. Statistical comparisons were performed with unpaired Student’s t-test (n = 3). *p < 0.05, **p < 0.01 versus A549. **(D, F)** The expression levels and quantitative analysis results of MMP2, MMP9, and EMT markers after exposure to DHW-221 at 48 h were tested by Western blot. **(E)** Immunofluorescence staining of vimentin and occludin in A549/Taxol cells were observed by a confocal microscopy, scale bar = 100 µm. Green, red, and blue fluorescence separately represented vimentin, occludin, and DAPI. The mean fluorescence intensity of vimentin and occludin were measured by ImageJ, and the relative fluorescence (green or red/DAPI ratio) were analyzed using GraphPad Prism 7.0. Statistical comparisons were carried out with one-way ANOVA followed by Dunnett’s *post-hoc* test for multiple comparisons (n = 3). Data are presented as mean ± SD. *p < 0.05, **p < 0.01, ***p < 0.001, ****p < 0.0001 versus control.

Based on the inhibitory effect of DHW-221 on the migration and invasion ability of NSCLC cells (24), we hypothesized that DHW-221 may also have a similar effect in A549/Taxol cells. A wound-healing assay was performed to verify the effects of DHW-221 on the migration capability of A549/Taxol cells. To exclude the effect of cell proliferation, we selected the concentration of DHW-221 that had no evident cytotoxic effect on A549/Taxol cells for subsequent experiments. As shown in **Figure 6A**, the wound-healing rate of DHW-221-treated A549/Taxol cells was significantly decreased compared to the control group, and similar results were found in A549 cells. In addition, the effect of DHW-221 on the cell invasion capability was further determined with or without Matrigel-coated Transwell membranes. After 24 h of DHW-221 treatment, the number of cells that migrated and invaded through the chamber was significantly reduced compared to the control group in DHW-221-treated A549/Taxol cells. These results suggested that DHW-221 inhibits the migration and invasion capabilities of A549/Taxol cells in a concentration-dependent manner (**Figures 6A, C**).

Extensive studies have shown that FOXO3a is involved in regulating the epithelial–mesenchymal transition (EMT) process in numerous cancers (43, 44). To verify whether A549/Taxol cells acquire the EMT phenotype, we examined the expression levels of epithelial markers (E-cadherin and occludin) and a mesenchymal marker (N-cadherin) in A549/Taxol and A549 cells. In A549/Taxol cells, the expression levels of E-cadherin and occludin were decreased compared to A549 cells, but the expression levels of N-cadherin were increased compared to A549 cells (**Figure 6B**), indicating that A549/Taxol cells have a stronger capacity for cell motility than A549 cells. The slender and fibrous morphology change in A549/Taxol cells (**Figure 1B**), which is a typical characteristic of a mesenchymal cells, suggested that A549/Taxol cells possess an EMT phenotype. To determine whether DHW-221 inhibits cell migration and invasion *via* the EMT process, the effect of DHW-221 on the expression of EMT-related proteins was examined by Western blot analysis. As shown in **Figure 6D**, DHW-221 treatment increased the expression levels of E-cadherin and occludin but significantly decreased the expression levels of mesenchymal markers, including vimentin and slug. Moreover, DHW-221 treatment decreased the expression of snail, which is a key transcription factor in regulating EMT (45). Matrix metalloproteinase (MMP) family proteins, such as MMP2 and MMP9, are of vital importance in cancer metastasis, and MMPs are highly expressed in cancers (46). Moreover, the expression levels of MMP2 and MMP9 were significantly downregulated by DHW-221 in A549/Taxol cells (**Figure 6D**). To further determine whether DHW-221 reverses EMT phenotypic changes, the expression of occludin and vimentin was visualized by immunofluorescence staining. With the increasing concentration of DHW-221, the green fluorescence intensity of vimentin was gradually weakened, whereas the red fluorescence intensity of occludin was enhanced in A549/Taxol cells (**Figure 6E**). Collectively, these findings suggested that DHW-221 suppresses the migration and invasion capabilities of A549/Taxol cells through reversing EMT phenotypic changes.

### DHW-221 Has Superior Antitumor Effects With no Obvious Toxicity in an Orthotopic A549/Taxol Tumor Mouse Model

**Figure 7:**
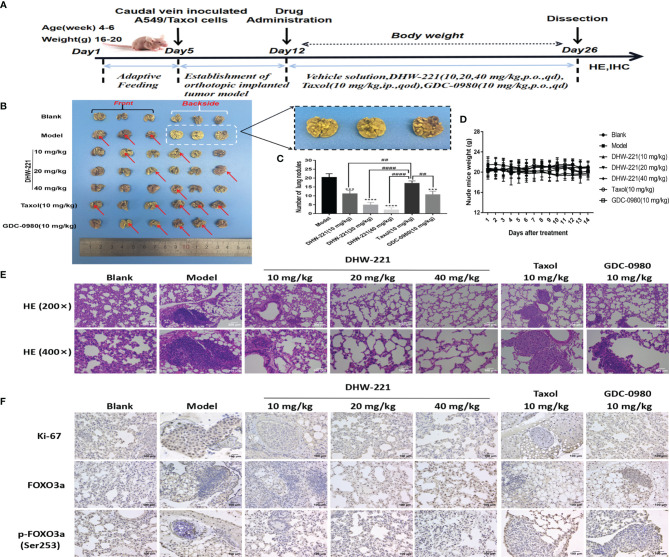
DHW-221 inhibits tumor growth in an orthotopic A549/Taxol nude mice model by tail vein. **(A)** The schematic figure of tumor modeling nude mice and drug administration. **(B)** Front and back images of orthotopic A549/Taxol lung samples in nude mice based on the Bouin’s staining (n = 6). Arrows and enlarged parts indicated nodules formed in the lungs. **(C)** Statistical analysis of the number of lung nodules in mice in each group (n=6). Statistical comparisons were performed with one-way ANOVA followed by Dunnett’s *post-hoc* test for multiple comparisons. Data were presented as mean ± SD. ***p < 0.001, ****p < 0.0001 versus control. ^##^p < 0.01, ^####^p < 0.0001 versus taxol. **(D)** Body weight change curve of nude mice after DHW-221, GDC-0980, and Taxol administrations. **(E)** Hematoxylin and eosin (HE) staining of lung tissues in each group under different magnifications (200× and 400×). Scale bar = 200 and 100 µm. **(F)** The expressions of Ki67, FOXO3a, and p-FOXO3a in lung tissues were detected by immunohistochemistry. Scale bar = 100 µm.

To further assess whether DHW-221 has antitumor activity in Taxol-resistant lung neoplasms, A549/Taxol cells were injected into nude mice *via* the tail vein to establish an orthotopic lung tumor model. After 1 week, DHW-221 (10, 20, and 40 mg/kg) and GDC-0980 (10 mg/kg) were intragastrically administered daily for 2 weeks, whereas Taxol (10 mg/kg) was intraperitoneally administered every 2 days (47, 48) (**Figure 7A**). Compared to the model and Taxol groups, the number of lung nodules was significantly decreased in the DHW-221-treated group (**Figures 7B, C**). To observe the pathological changes of mouse lung tissues, HE staining was performed. Compared to the blank group, the alveoli of the model and Taxol groups were covered by nodules, while the lung nodule formation in the DHW-221 and GDC-0980 group was slower than that of the model and Taxol groups (**Figure 7E**). Of note, we observed sparse pink cytoplasm and densely stained round or oval vesicular nuclei in tumor cells in the model and Taxol groups (**Figure 7E**). Additionally, to verify whether DHW-221 induces FOXO3a translocation *in vivo*, we examined the expression levels of FOXO3a and p-FOXO3a by IHC. After DHW-221 treatment, p-FOXO3a expression was downregulated, and FOXO3a expression was upregulated (**Figure 7F**). Ki67 is an indicator of proliferative capacity in cancers, and Ki67 expression is closely associated with tumor invasion and metastasis; thus, Ki67 expression affects the prognosis of cancer patients (49). In the present study, we examined Ki67 expression by IHC. Compared to the blank group, Ki67 expression was increased in the model and Taxol groups, but there was no change in Ki67 expression in the DHW-221 and GDC-0980 groups (**Figure 7F**). These findings suggested that DHW-221 inhibits tumor growth through FOXO3a translocation in A549/Taxol cell-bearing mice.

**Figure 8:**
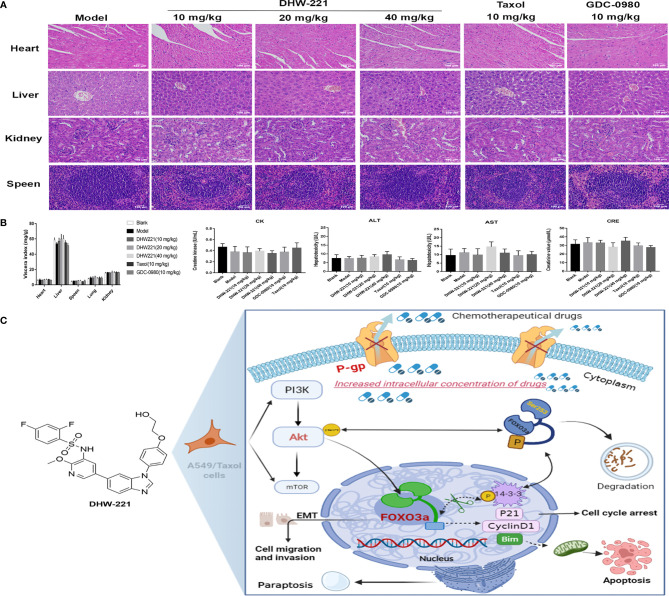
DHW-221 has no obvious toxicity in DHW-221-administered nude mice. **(A)** HE staining of the heart, liver, kidney, and speen from orthotopic nude mice. Scale bar = 100 µm. **(B)** The viscera index of organs (heart, liver, speen, lung, and kidney) and the biochemical parameters of heart (CK), liver (ALT, AST), and kidney (CRE) from the serum of each group in orthotopic nude mice. Data were presented as mean ± SD. Statistical comparisons were carried out with one-way ANOVA followed by Dunnett’s *post-hoc* test for multiple comparisons (n = 6). **(C)** Molecular mechanism underlying the anticancer effects of DHW-221 on the P-gp and PI3K/Akt/FOXO3a signaling pathway in A549/Taxol cells.

Although some chemotherapeutic drugs have high efficacy in clinic, many are limited due to their toxicity. In the present study, we evaluated the toxicity of DHW-221 *in vivo*. During treatment, there were no significant differences in the body weight and viscera index between the DHW-221 and GDC-0980 groups in A549/Taxol cell-bearing mice (**Figures 7D**, **8B**). Moreover, after DHW-221 treatment, the model mice did not show any behavioral abnormalities or loss of appetite. Intriguingly, a mouse in the model, Taxol, and 10 mg/kg DHW-221 A549/Taxol group died of hind limb paralysis after being treated with drugs for 14 days, which may have been caused by overburden of the tumor compressing the nerves in the hind limbs. Evaluation of the histological changes in the visceral organs due to DHW-221 treatment in A549/Taxol cell-bearing mice by H&E staining demonstrated that there were no evident histological abnormalities in myocardial tissue, hepatocyte morphology, glomeruli, and splenic corpuscles (**Figure 8A**). To further determine the toxicity of DHW-221, biochemical parameters, including CK, ALT, AST, and CRE, were evaluated in the heart, liver, and kidney. After DHW-221 treatment, there were no abnormal toxicity signs in the CK, ALT, AST, and CRE levels in A549/Taxol cell-bearing mice (**Figure 8B**). Taken together, these results suggested that DHW-221 suppresses tumor growth without body weight change and toxicity through FOXO3a translocation *in vivo*.

## Discussion

Lung cancer has been a serious threat to human health worldwide due to high morbidity and mortality, especially for NSCLC (50). Paclitaxel, as a first-line chemotherapeutic drug, is widely used to treat NSCLC in the clinic (5). However, acquired resistance to Taxol greatly affects its clinical efficacy and patient prognosis during treatment, especially due to the emergence MDR (51). MDR in cancer continues to be an inevitable phenomenon that limits the efficacy of chemotherapeutical drugs against NSCLC (52), in which cancer cells gain the capacity to develop cross-resistance to a wide range of structurally and functionally irrelevant drugs. Therefore, it is important to understand how to overcome MDR.

Previously, we designed and synthesized DHW-221, as a dual PI3K/mTOR inhibitor, and we showed that it exerts a potent antitumor activity against NSCLC cells ***in vitro*** and ***in vivo*** (23, 24). In the present study, we found that A549/Taxol cells gained cross-resistance to Taxol, docetaxel, vincristine, and cisplatin, resulting in elongated and fibroid morphology (**Figure 1B**). We also demonstrated that DHW-221 suppressed cell proliferation and colony formation in a concentration- and time-dependent manner in A549/Taxol cells compared to A549 cells and that this affect was superior to that of GDC-0980 (a dual PI3K/mTOR inhibitor) (**Figures 1D, E**). Therefore, we speculated that DHW-221 may represent a valuable therapeutic agent in Taxol-sensitive and Taxol-resistant NSCLC therapy. We identified the underlying molecular mechanisms by which DHW-221 exhibits an excellent inhibitory potency to conquer MDR in A549/Taxol cells.

P-gp is considered as an energy-dependent drug discharge pump that mediates MDR by decreasing intracellular drug accumulation (9). Previous studies have shown that P-gp is overexpressed in several MDR cancer cells (9, 53), which prompted us to develop a novel P-gp inhibitor to successfully avoid the risks of drug efflux (13, 54). Rhodamine 123 (Rho-123) acts as a P-gp substrate and is used to measure P-gp function (26). In the present study, we found that DHW-221 and verapamil (a known first-generation P-gp inhibitor) significantly increased the intracellular accumulation of Rho-123 in A549/Taxol cells (**Figure 2B**). Additionally, chemotherapeutics in combination with a P-gp inhibitor is also an alternative approach to achieve more durable therapeutic effects in MDR cancer cells (55, 56). Treatment with 50 nM DHW-221 significantly potentiated the efficacy of Taxol in A549/Taxol cells (**Figure 2D**). Moreover, the overexpression of P-gp was significantly reversed by DHW-221 in A549/Taxol cells (**Figure 2E**), implying that DHW-221 may be a P-gp inhibitor and a MDR reversal agent to inhibit P-gp expression and function. These results supported the results of previous studies wherein BZML inhibits P-gp function, thereby conferring A549/Taxol cell MDR; however, it remains unclear whether BZML binds to ABCB1 (47). In contrast, the present study further verified that DHW-221 enhanced ABCB1 protein stability to inhibit its expression and function ***via*** binding to ABCB1 (**Figures 2F, G**). Furthermore, Zhang et al. reported that inhibition or knockout of PI3K 110α or 110β overcomes P-gp-mediated MDR in cancer (22). Therefore, these characteristics may contribute, at least in part, to the ability of DHW-221 to overcome MDR.

Apoptosis induced by the mitochondrial endogenous pathway and mitotic arrest has become a common cell death mode of many anticancer drugs in clinic. As expected, DHW-221 augmented the pro-apoptotic effect through activating the caspase-dependent mitochondrial pathway and inducing cell cycle arrest at the G0/G1 phase in A549/Taxol cells (**Figures 3A–D**, **4C**), which agreed with a previous study (24). However, apoptosis evasion tends to make tumor cells to be more resistant to chemotherapy drugs (57, 58), which is frequently caused by overexpression of antiapoptotic Bcl-2 family proteins (59, 60). Su et al. demonstrated that a Bcl-2 inhibitor may represent a promising and alternative agent for the treatment of Bcl-2 overexpressed refractory or recurrent hematological malignancies when conventional chemotherapy fails (61). In the present study, DHW-221 significantly downregulated Bcl-2 expression in a concentration-dependent manner (**Figure 3D**), suggesting that DHW-221 triggers cell apoptosis through the activating caspase-dependent mitochondrial pathway in a manner dependent on Bcl-2 expression.

Accumulating evidence has shown that reactivating cell apoptosis and cell cycle arrest are not efficient strategies to improve the efficacy of chemotherapeutics (31). Thus, it is highly desirable to develop novel anticancer agents that are independent of cell apoptosis to overcome chemoresistance in cancers (31, 62). Paraptosis is a non-apoptotic and caspase-independent form of programmed cell death, and it is typically characterized by cytoplasmic vacuolation derived from the ER (30, 63). Emerging evidence has highlighted that paraptosis is an important and alternate cell death pathway for overcoming chemoresistance, including MDR, in human cancer cells (31, 64). Chen et al. reported that curcuminoid B63 induces paraptosis-like cell death in 5-fluorouracil (5-FU)-resistant gastric cancer cells. Moreover, Li et al. found that honokiol reverses Taxol resistance by inducing paraptosis in H1650 cells (65). Interestingly, the present study demonstrated that high-concentration DHW-221 (2.4 μM) significantly triggered cell paraptosis *via* activating ER stress and the MAPK signaling pathway in A549 cells (data not shown). These findings were consistent with previous studies showing that high concentration of Taxol induces paraptosis-like cell death *via* an ER vacuolization-mediated pathway in A549 cells (66). Taken together, these findings indicated that DHW-221 triggers three diverse cell death modes, namely, apoptosis, paraptosis, and cell cycle arrest, which may amplify the cytotoxic effect of DHW-221 in MDR A549 cells.

Abnormal activation of the PI3K/Akt signaling pathway is strongly linked to the MDR in cancer (67), including Taxol-resistant breast (68) and prostate cancers (69). Our results demonstrated that abnormal activation of the PI3K/Akt signaling pathway was significantly inhibited by DHW-221 in A549/Taxol cells (**Figures 5A, B**). In addition, Forkhead box O3a (FOXO3a), an important tumor transcription factor in the FOXO family and a direct downstream target of Akt, is essential for regulating multiple physiological processes, including cell proliferation, cell apoptosis, migration, and longevity (14). In the clinic, the cytostatic and cytotoxic effects of various anticancer drugs, including paclitaxel, doxorubicin, lapatinib, imatinib, and cisplatin, are mediated by FOXO3a activation (70). Borris et al. revealed that the involvement of FOXO3a activity in regulating P-gp activity response involves doxorubicin-induced PI3K/Akt signaling (71). The present study demonstrated that PI3K/Akt-mediated FOXO3a overactivation caused MDR in A549/Taxol cells (**Figure 5B**). Cytoplasmic translocation of Akt-dependent FOXO3a has emerged as a key process in carcinogenesis for evading cancer cell apoptosis (15, 72). The Bcl-2 subfamily member and BH3-only protein, Bim, is activated when cells receive intrinsic apoptosis signals, which inhibits the activity of the antiapoptotic protein, Bcl-2, thereby inducing the release of proapoptotic proteins and ultimately leading to permeabilization of the mitochondrial outer membrane (73). Furthermore, FOXO3a regulates Bim expression at the transcriptional level (42). In the present study, DHW-221 significantly increased the expression levels of FOXO3a and Bim but inhibited the expression levels of Bcl-2 in A549/Taxol cells (**Figures 3D**, **5B**). Methylseleninic acid has been reported to induce cell apoptosis *via* Akt-mediated nuclear FOXO3a translocation in A549 cells (74). In the present study, we demonstrated that DHW-221 increased FOXO3a expression and transferred it from the cytoplasm to the nucleus in A549/Taxol cells (**Figures 5C–E**). Previous studies have reported that Akt activation stimulates FOXO3a degradation *via* the proteasome pathway (17). The present study further confirmed that DHW-221 interfered with FOXO3a degradation in a proteasome-dependent manner (**Figures 5F, G**). Consistent with *in vitro* studies, DHW-221 significantly suppressed tumor growth without significant toxicity and body weight changes through FOXO3a nuclear translocation *in vivo* (**Figures 7**, **8A, B**). Collectively, these findings suggested that mitochondrial apoptosis induced by DHW-221 is regulated by Akt-mediated FOXO3a nuclear translocation in A549/Taxol cells.

In addition, nuclear translocation of FOXO3a also contributes to cell cycle arrest through activating transcriptional targets, such as p21^Cip1^ (p21) and cyclin D1 (75). The cyclin D1-CDK4/6 complex plays a key role in the cell cycle process, as it phosphorylates and inactivates Rb protein, thereby blocking the cell proliferation cycle in S phase (38, 76). Song et al. found that Tic10 arrests at the G0/G1 phase by decreasing the expression levels of FOXO3a-dependent CDK4/6 proteins in 5-FU-resistant breast cancer cells (77). The present study demonstrated that DHW-221 upregulated p21 protein expression and downregulated cyclin D1, CDK4, and CDK6 protein expression *via* FOXO3a-mediated mechanisms, thereby inducing cell cycle arrest at the G0/G1 phase and apoptosis in A549/Taxol cells (**Figures 4C, D**). These findings indicated that the DHW-221-induced G0/G1 phase arrest participates in the cell death process in A549/Taxol cells.

EMT is highly involved in tumor invasion and metastasis during tumorigenesis and development. Studies have suggested that EMT is one of most common causes of chemotherapy drug resistance (78). Li et al. reported that acquired drug resistance is related to EMT and that drug-resistant cells exhibit stem cell-like properties (79). In the present study, we found that the A549/Taxol cells showed morphological changes with a mesenchymal-like phenotype and higher cell motility (**Figures 1B**, **6B**), which was consistent with our previous study (80). EMT is characterized by loss of the epithelial cell adhesion markers, E-cadherin and occludin, and the upregulation of mesenchymal cell-associated proteins, such as N-cadherin, vimentin, and snail. Previous studies have reported that blockade of the PI3K/Akt/mTOR signaling pathway alleviates ovarian cancer chemoresistance through reversing the EMT process. The present study demonstrated that DHW-221 significantly suppressed the invasion and metastasis of A549/Taxol cells by regulating the expression of EMT-related proteins *in vitro* (**Figures 6A–F**). Ki67, a nuclear proliferation-related protein, is closely associated with tumor invasion and metastasis in cancers (49). In the present study, Ki67 expression was reduced after DHW-221 treatment in the MDR A549 nude mouse model, further confirming the role of DHW-221-induced inhibition of invasion and migration *in vitro* and *in vivo*. Taken together, these results provided molecular evidence that the PI3K/mTOR dual inhibitor, DHW-221, overcomes MDR *via* targeting P-gp and Akt-mediated FOXO3a nuclear translocation in NSCLC.

The present study had several limitations. First, FOXO3a has been widely acknowledged to affect cell invasion and metastasis through regulating the EMT process in numerous cancer cells (14, 81), but the relationship between the two is rarely reported in drug-resistant cells (82). Unfortunately, the present study lacked experimental evidence to clarify the molecular mechanism of the DHW-221-mediated regulation of the EMT process *via* FOXO3a nuclear translocation in A549/Taxol cells. Second, there was no significant difference in FOXO3a expression and Bim expression in A549 cells (**Figure 5B**), implying that cell apoptosis triggered by DHW-221 is not regulated by Akt-mediated FOXO3a in A549 cells. However, this mechanism needs to be further confirmed by experiments in future work. Third, in an established human orthotopic A549/Taxol mouse tumor model *via* tail vein, the initial randomization of mice does not base on the tumor volume/size of each mouse, although we count the number of lung nodules to evaluate tumor growth in each group; the tumor growth inside the lung is possibly ignored. We think that it may not be the optimal way to assess tumor growth of mice *in vivo*. Thus, our future studies will further clarify the underlying mechanisms of DHW-221 as a MDR reversal agent in MDR A549 cells by the *in vivo* imaging technology of small animals to provide more evidence for reversal of Taxol-resistance to help solve the drug-resistance problem.

In conclusion, the present study for the first time demonstrated that DHW-221, as a PI3K/mTOR dual inhibitor and novel P-gp inhibitor, exerts potent cytotoxic activity and induces cell apoptosis to overcome MDR through Akt-mediated FOXO3a nuclear translocation in NSCLC (**Figure 8C**, made in https://app.biorender.com/) both *in vitro* and *in vivo*. Moreover, DHW-221 induces cell cycle arrest and paraptosis *via* activating ER stress and MAPK signaling. In addition, DHW-221 suppresses cell migration and invasion by reversing the EMT process. Therefore, DHW-221 represents a prospective therapeutic candidate for further investigation in drug-resistant NSCLC therapy. The present study provided information for a PI3K/mTOR dual inhibitor, which may aid in solving the drug resistance in NSCLC.

## Data Availability Statement

The original contributions presented in the study are included in the article/**Supplementary Material**. Further inquiries can be directed to the corresponding authors.

## Ethics Statement

The animal study was reviewed and approved by Ethics Committee for Animal Experiments of Shenyang Pharmaceutical University.

## Author Contributions

ML: conceptualization, methodology, investigation, formal analysis, writing—original draft. CX and XQ: resources, validation, supervision, writing—review and editing. WL, DL, HJ, and XG: resources, software, writing—review and editing. YW, QW, XX, and BX: resources and validation. XJ and HL: validation and supervision. YZ and HD: supervision. QZ: conceptualization, data curation, writing—review and editing, project administration, and funding acquisition. All authors contributed to the article and approved the submitted version.

## Funding

This study was supported by Liaoning Natural Fund Guidance Plan (Number 2019-ZD-0446) and State Key Laboratory of Chemical Oncogenomics, Tsinghua Shenzhen International Graduate School.

## Conflict of Interest

The authors declare that the research was conducted in the absence of any commercial or financial relationships that could be construed as a potential conflict of interest.

## Publisher’s Note

All claims expressed in this article are solely those of the authors and do not necessarily represent those of their affiliated organizations, or those of the publisher, the editors and the reviewers. Any product that may be evaluated in this article, or claim that may be made by its manufacturer, is not guaranteed or endorsed by the publisher.

## Abbreviations

NSCLC, non-small cell lung cancer; MDR, multidrug resistance; Taxol, Paclitaxel; ABC, ATP-binding cassette; P-gp, P-glycoprotein; ABCB1, ABC subfamily B member 1; PI3K, phosphatidylinositol-3-kinase; FOXO3a, Forkhead box O3a; MTT, 3-(4,5-dimethylthiazol-2-yl)-2,5-diphenyltetrazolium bromide; Rho-123, Rhodamine; DAPI, 4,6-diamino-2-phenyl indole; HE, hematoxylin and eosin; IHC, immunohistochemistry; EMT, epithelial–mesenchymal transition; μM, micromolar; μm, micrometer; IC50, 50% inhibitory concentration; p-FOXO3a, phosphorylated FOXO3a; p-Akt, phosphorylated Akt.
