# Supplementary material for: DHW-221, a Dual PI3K/mTOR Inhibitor, Overcomes Multidrug Resistance by Targeting P-Glycoprotein (P-gp/ABCB1) and Akt-Mediated FOXO3a Nuclear Translocation in Non-small Cell Lung Cancer
Source: Front Oncol. 2022 May 13;12:873649. doi: 10.3389/fonc.2022.873649 (PMC9137409; doi:10.3389/fonc.2022.873649)
Supplement: Supplementary file 1 [file DataSheet_1.docx]

| Treatment time | IC_50_(μM) | |
| --- | --- | --- |
|  | A549 | A549/Taxol |
| 24 h | > 2.400 | > 2.400 |
| 48 h | 0.631 ± 0.072 | 0.874 ± 0.056 |
| 72 h | 0.424 ± 0.024 | 0.527 ± 0.034 |

**Table S1** The inhibitory activity for DHW-221 at 24 h, 48 h and 72 h in A549 and A549/Taxol cells (mean ± SD, n=3).


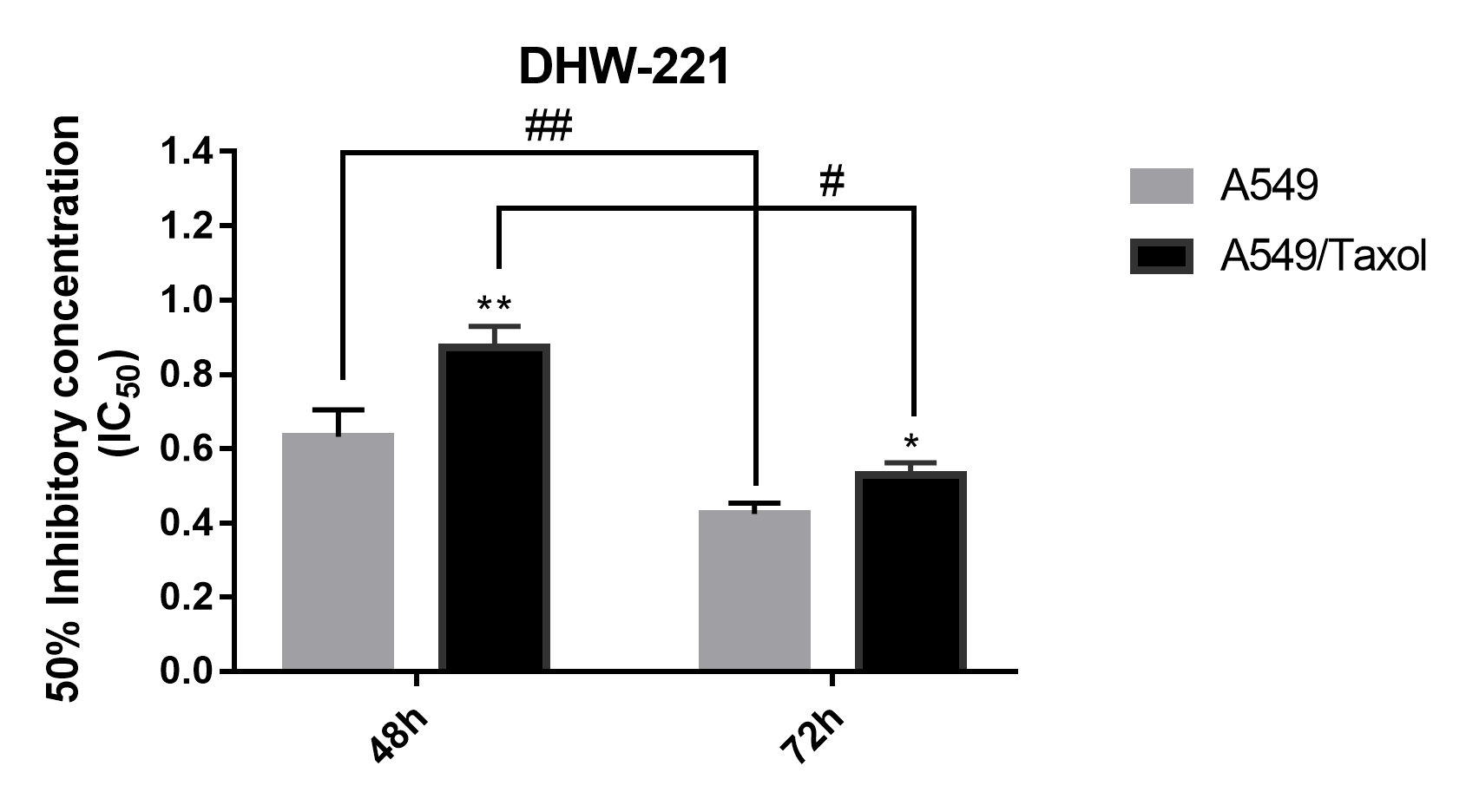


Figure S1. The statistical result of IC_50_ values of DHW-221 at different times in A549 and A549/Taxol cells. Data were presented as mean ± SD. *p < 0.05, **p < 0.01 versus A549. ^#^p < 0.05,  ^##^p < 0.01 versus 48h.
